# Functional Cloning and Expression of the *Schizophyllum commune* Glucuronoyl Esterase Gene and Characterization of the Recombinant Enzyme

**DOI:** 10.1155/2012/951267

**Published:** 2012-07-04

**Authors:** Dominic W. S. Wong, Victor J. Chan, Amanda A. McCormack, Ján Hirsch, Peter Biely

**Affiliations:** ^1^Western Regional Research Center, USDA-ARS, Albany, CA 94710, USA; ^2^Institute of Chemistry, Slovak Academy of Sciences, Dúbravská Cesta 9, 845 38 Bratislava, Slovakia

## Abstract

The gene encoding *Schizophyllum commune* glucuronoyl esterase was identified in the scaffold 17 of the genome, containing two introns of 50 bp and 48 bp, with a transcript sequence of 1179 bp. The gene was synthesized and cloned into *Pichia pastoris* expression vector pGAPZ*α* to achieve constitutive expression and secretion of the recombinant enzyme in soluble active form. The purified protein was 53 kD with glycosylation and had an acidic pI of 3.7. Activity analysis on several uronic acids and their derivatives suggests that the enzyme recognized only esters of 4-*O*-methyl-D-glucuronic acid derivatives, even with a 4-nitrophenyl aglycon but did not hydrolyze the ester of D-galacturonic acid. The kinetic values were *K*_*m*_ 0.25 mM, *V*_max_ 16.3 *μ*M·min^−1^, and *k*_cat_ 9.27 s^−1^ with 4-nitrophenyl 2-*O*-(methyl 4-*O*-methyl-*α*-D-glucopyranosyluronate)-*β*-D-xylopyranoside as the substrate.

## 1. Introduction

In the current schemes of biomass conversion, pretreatment with enzyme hydrolysis recovers only about 85% of the theoretical yield for the available sugars [1]. Development of a cost-competitive process is hampered by the lack of knowledge on the breakdown of covalent cross-linkages connecting cellulose, hemicellulose, and lignin in plant cell walls. As much as 90% of the lignin in woody plants might be covalently linked to polysaccharides [2]. The types of covalent lignin-carbohydrate linkages have been proposed to include lignin alcohol esters, ethers, and phenyl glycosides [3–5]. The wood-rotting fungus *Schizophyllum commune* has been shown to produce a glucuronoyl esterase (*Sc*GE), which cleaves substrate mimics of ester bonds between lignin alcohols and glucuronoxylan [6]. Other carbohydrate esterases, acetylxylan esterases, feruloyl esterases, and pectin methylesterases, did not act on these substrates. GE enzymes were subsequently isolated from other source microorganisms, including *Hypocrea jecorina*, *Phanerochaete chrysosporium*, and *Sporotrichum thermophile* [7–9]. In this paper, the putative cDNA gene of glucuronoyl esterase in the genome of the original source microorganism, *Schizophyllum commune* was identified, synthesized, cloned, and expressed in *Pichia pastoris*. The recombinant enzyme (r*Sc*GE) was purified and its enzyme action characterized on uronic acid substrates and their derivatives.

## 2. Materials and Methods

### 2.1. Materials and Strains

The pGAPZ*α*-A vector, strain SMD1168, Zeocin, protein extraction reagent, precast gel, protein standards and staining kits were purchased from Invitrogen (San Diego, CA, USA). Gene DNA synthesis was performed by EZBiolab (Carmel, IN, USA), and primers and oligos were synthesized by Elim Biotech (Hayward, CA, USA). Restriction enzymes and DNA modifying enzymes were obtained from New England Biolab (Beverly, MA, USA). Plasmid prep, DNA extraction, and PCR minicolumns were obtained from Qiagen (Valencia, CA, USA). Ni-Sepharose resin was purchased from GE Healthcare (Piscataway, NJ, USA). Coomassie Plus Protein Assay Reagent, N-glycosidase F, and Glycoprotein Deglycosylation Kit were purchased from Pierce (Rockford, IL, USA).

### 2.2. Gene-Vector Construction

The *Sc*GE gene was synthesized to include 1179 nucleotides of the CDS (encoding all 393 amino acids except the start Met), a 5′*Eco*RI site and a 3′*Xba*I site. The gene was cloned into *Pichia pastoris* expression vector pGAPZ*α*-A by restriction digest and ligation using standard methods. For transformation, the DNA-vector construct was linearized at the unique *Bsp*HI site, gel-isolated, and recovered.

### 2.3. Transformation of Pichia pastoris

Competent yeast cells were prepared using the EasyComp Kit (Invitrogen). For each transformation, 2 *μ*g of linearized DNA was mixed with 50 *μ*L of competent cells of SMD1168, a protease-deficient strain of *Pichia pastoris*, and chemically transformed following the manufacturer's protocol. Transformants were plated on YPD agar plates containing 1000 *μ*g/mL Zeocin antibiotic and incubated at 30°C for three days. All colonies were restreaked on YPD Zeocin to obtain pure isolates. 

### 2.4. Expression and Purification of the Recombinant Glucuronoyl Esterase

A single yeast colony was used to inoculate 10 mL of buffered complex 2% glucose media (1% yeast extract, 2% peptone, 100 mM potassium phosphate, pH 6.0, 1.34% yeast nitrogen base, 4 × 10^−5^% biotin, and 2% glucose) and shaken at 225 rpm and 30°C overnight. Overnight culture of 5 mL was diluted into 500 mL of media (1 : 100) in a 2-liter baffled flask and shaken at 225 rpm and 30°C for 5 days. The culture was centrifuged 10 min at 10,000 ×g and passed through a 0.45 *μ*m polyethylene sulfone filter. The clarified culture supernatant was concentrated using a PelliconXL device with a Biomax10 membrane on a Labscale TFF pump (Millipore) and buffer exchanged to 0.3 M NaCl, 50 mM Na phosphate, pH 7.0. The concentrated supernatant was applied to an AKTA prime FPLC fitted with a HisTrap HP column at a flow rate 0.3 of mL/min and eluted in a linear gradient of 0 to 200 mM imidazole over 30 mL. The protein peak was eluted approximately between 50 and 125 mM imidazole. Fractions were analyzed on a 4–12% Bis-Tris NuPAGE run at 200 V 50 min. Pooled fractions were concentrated and buffer exchanged to 10% glycerol, 25 mM Na phosphate, pH 6.0 buffer. 

### 2.5. Enzyme Activity Assay

**Figure 1:**
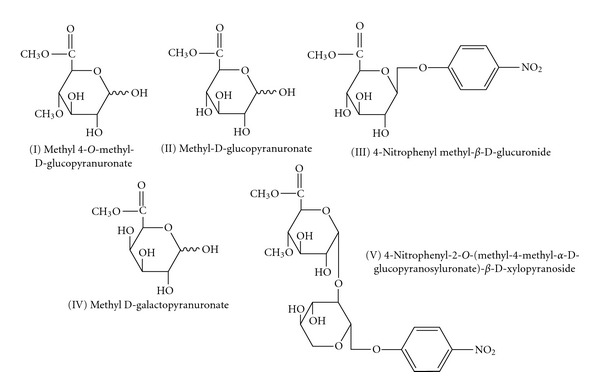
Substrate compounds used for activity and kinetic measurements [10].

A qualitative assay of glucuronoyl esterase by TLC was performed using the following substrates: (I) methyl 4-*O*-methyl-*α*-D-glucopyranuronate, (II) methyl D-glucopyranuronate, (III) 4-nitrophenyl methyl-*β*-D-glucuronide, methyl D-galactopyranuronate, and (IV) methyl-D-galacturonate (Figure 1). Enzyme reactions were performed by incubating 1 *μ*g of purified enzyme with 20 mM of substrate in 50 mM Na phosphate buffer, pH 6.0, at 30°C for 20 min. The reaction mixture was spotted on Silica gel 60 TLC plates developed in ethyl acetate : acetic acid : 1-propanol : formic acid : water (25 : 10 : 5 : 1 : 15) and visualized with* N*-(1-naphthyl) ethylenediamine dihydrochloride) [10, 11].

The enzyme activity was quantified by HPLC using compound V: 4-nitrophenyl 2-*O*-(methyl 4-*O*-methyl-*α*-D-glucopyranosyluronate)-*β*-D-xylopyranoside as the substrate (Figure 1, structure V). The substrate was used at concentrations from 0.08 to 0.4 mM in 50 mM Na phosphate buffer, pH 6.0, with enzyme added at 0.157 *μ*M. Equal volume of cold 1 M Na acetate, pH 4.5 was added to stop the reaction, and the sample was analyzed by a HPLC system fitted with a UV detector at 300 nm using a C18 column with water : formic acid : acetonitrile (7 : 1 : 2) as the solvent at a flow rate of 0.3 mL/min. The kinetic values were obtained by nonlinear regression analysis of the Michaelis-Menten plot of *v* as a function of [s]. A Lineweaver-Burk plot was derived from transformation based upon nonlinear regression analysis that reflects the best possible estimates.

### 2.6. Deglycosylation

The r*Sc*GE protein was denatured by heating at 100°C for 10 min in SDS/*β*-mercaptoethanol Na phosphate buffer, pH 7.0, followed by addition of N-glycosidase F as recommended by the supplier of the glycosylation kit. The reaction mixture was incubated at 37°C for 1 hr, before running SDS-PAGE. For carbohydrate estimation, r*Sc*GE was oxidized by sodium metaperiodate to aldehydes, which reacted with glycoprotein detection reagent to form a purple product with maximum absorbance at 550 nm.

### 2.7. Bioinformatics

Vector NTI and Geneous were used for sequence analysis and construction. Homology modeling was accomplished by the use of Swiss Model [12]. GraphPad Prism 4 was used for kinetic analysis.

## 3. Results

The putative gene sequence (jgi Schcol 238770 fgenesh2_pg.17_#_99) for *Schizophyllum commune* glucuronoyl esterase was found to locate at scaffold 17 of the genome (MW_003315656), containing two introns of 50 bp and 48 bp, with a transcript (coding) sequence of 1179 bp (http://genome.jgi-psf.org/). A transcription element TATA box within the promoter region, and a polyadenylation sequence AACAAAA at ~200 bp downstream of the stop codon were identified. The structural gene encodes a protein of 393 amino acids, with a predicted molecular mass of 41.8 kD and a pI of 4.1. The N-terminal 19 residues form a signal peptide with the cleavage site predicted between Ala19 and Gln20. Extensive blast search of the *S. commune* genome with the highly conserved regions of the known GE enzymes did not reveal a second GE gene in the genome. 

**Figure 2:**
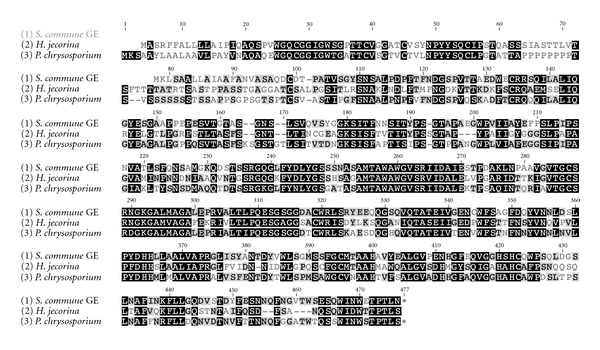
Multiple sequence alignment of *S. commune* (XP_003026289), *H. jecorina *(AAP57749), and *P. chrysosporium* (e_gwh2.18.77.1) glucuronoyl esterases using Geneious software.

The translated sequence of the identified *S.  commune* GE gene shows an exact match with the peptide sequence obtained by Edman analysis of the previously biochemically characterized *Sc*GE (DTPATVSGYSNSALPDPF). There is also an exact match with the internal tryptic peptide (AGALEPRVALTLPQE) of the native *Sc*GE enzyme as well [7]. Blast search revealed close alignment with, *H. jecorina* Cip2 (AAP57749) and *P. chrysosporiumge*1 (e_gwh2.18.77.1), showing similarities of 59.0, 67.1% and identities of 51.1, 61.0%, respectively (Figure 2). The *Sc*GE sequence also shows 56.5% similarity and 48.1% identity with the recently reported *S. thermophile* enzyme [9].

**Figure 3:**
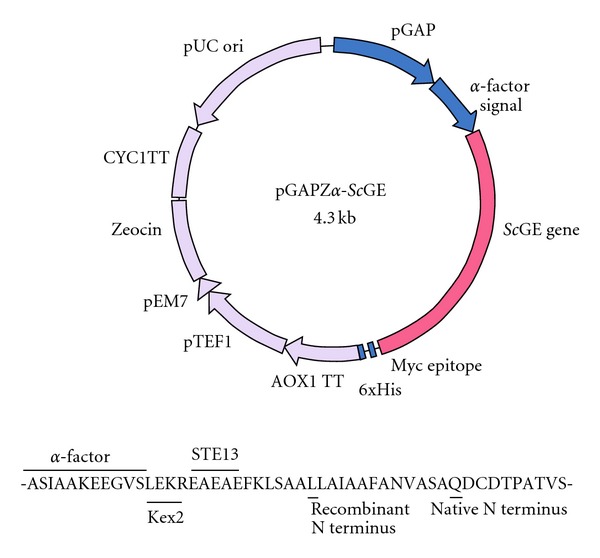
Construction scheme of the recombinant plasmid pGAPZ*α*-*Sc*GE. Below shows sequence details of the *α*-factor junction with the N-terminus.

The gene-vector *Sc*GE-pGAPZ*α* cloned into the *Pichia pastoris* for expression consisted of 1176 bp CDS (encoding all amino acids except the start Met), a 30 bp *myc* epitope tag, an 18 bp polyhistidine tag sequence, and a Zeocin resistance gene for selection (Figure 3). The total nucleotide sequence of the construct translates to a molecular mass of 41.6 kD. The gene vector was transformed into SMD1168, a protease deficient strain, for expression of the recombinant protein. The r*Sc*GE was purified from culture supernatant by ultrafiltration followed by Ni-Sepharose affinity chromatography. The purified protein showed an N-terminal sequence of LSAALLAIAAFA indicating that the vector *α*-factor signal was cleaved at three amino acid residues after the *Ste13* protease site (Figure 3).

**Figure 4:**
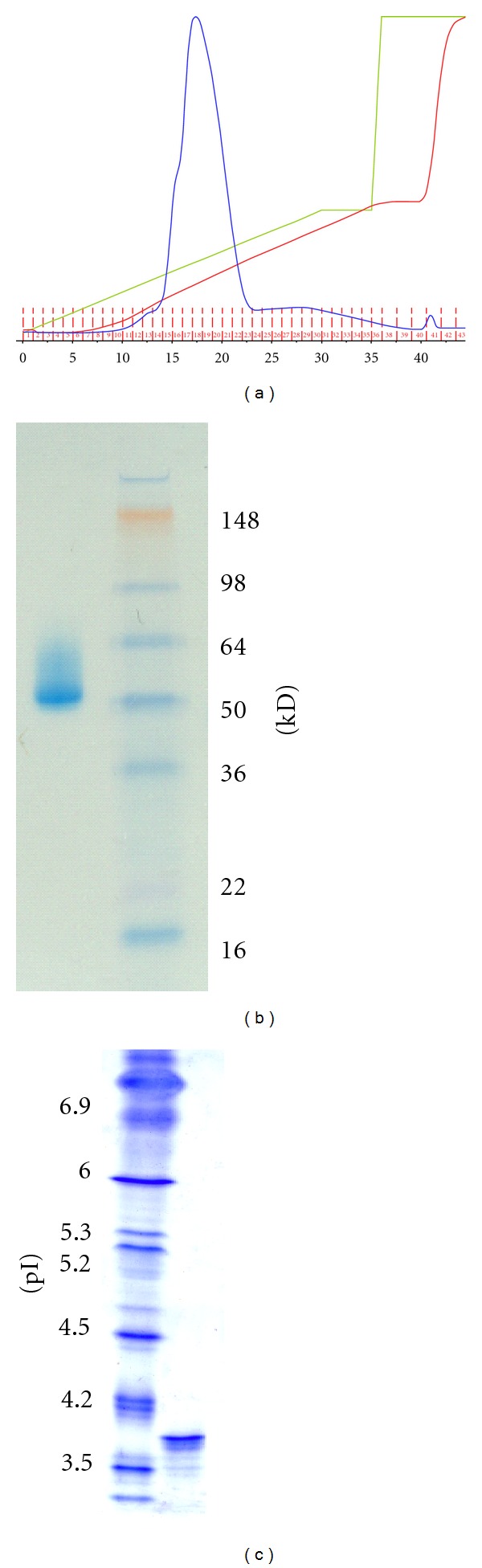
(a) Chromatogram of purification by Ni Sepharose column. Details described in “Methods” section. (b) SDS-PAGE on 40–12% Novex Bis-Tris gel. (c) IEF on Invitrogen pH 3–10 IEF gel.

**Figure 5:**
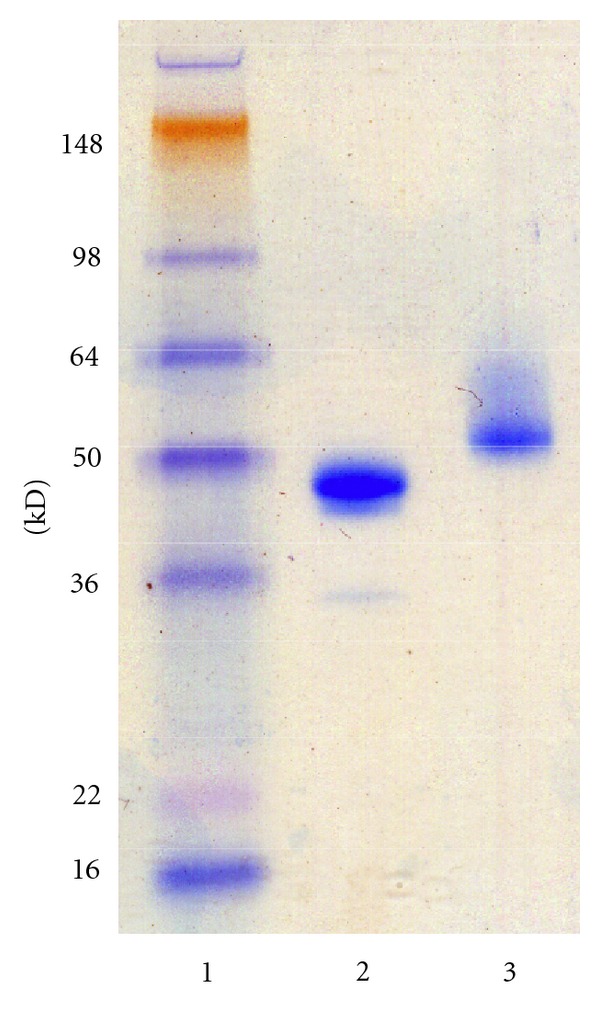
SDS-PAGE of r*Sc*GE (lane 3) and deglycosylated r*Sc*GE (lane 2). Deglycosylation reaction described in “Methods” section, performed on 4–12% Novex Bis-Tris gel, stained by Invitrogen Simply Blue SafeStain.

SDS-PAGE of r*Sc*GE showed a band ≥53 kD, which was larger than predicted from the nucleotide sequence (Figure 4). Isoelectrofocusing revealed that the r*Sc*GE was in multiple forms with the major band having a pI of 3.7. The band patterns on both the SDS-PAGE and IEF gels suggested that the protein was glycosylated. Treatment of r*Sc*GE with *N*-glycosidase-F caused the band sharpened and shifted in electrophoretic migration to 46 kD, closer to the calculated size (Figure 5). Results from periodate oxidation suggested that the protein contained ~1.4% carbohydrate content. 

**Figure 6:**
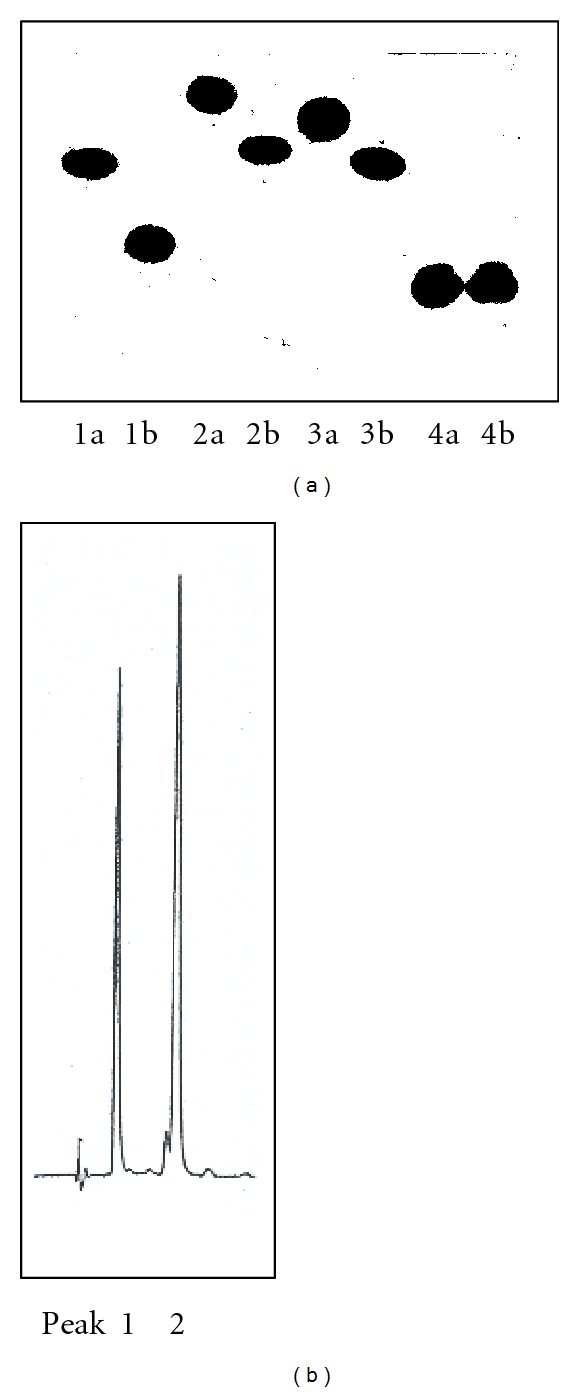
Separation of substrate and product components in reaction mixtures by (a) TLC: 1a, b (substrate I & product), 2a, b (II & product), 3a, b (III & product), 4a, b (IV & product); (b) HPLC: peak 1, 2 for the acid (product) and ester of substrate V, respectively. Reaction conditions: 20 mM substrate in 50 mM sodium phosphate buffer, pH 6.0, 1 *μ*g r*Sc*GE, 30°C, 30 min. See details on TLC and HPLC runs in “Methods” section.

The r*Sc*GE enzyme tested for its activity on methyl esters of uronic acids and their glycosides (substrates I to V, Figure 1) showed catalytic properties using compounds I–V as substrates. The enzyme reactions were performed at pH 6.0 as the highest value, because the ester linkages become unstable under more alkaline conditions. The enzyme action on substrates I to IV was monitored by TLC, based on the migration *R*_*f*_ difference between the methyl ester substrates and the deesterified product (Figure 6(a)). The enzyme reaction using substrate V was analyzed by HPLC (Figure 6(b)). The enzyme was not active on substrate IV, which contained an esterified galacturonic acid moiety instead of glucuronic acid as in the other four substrates.

**Figure 7:**
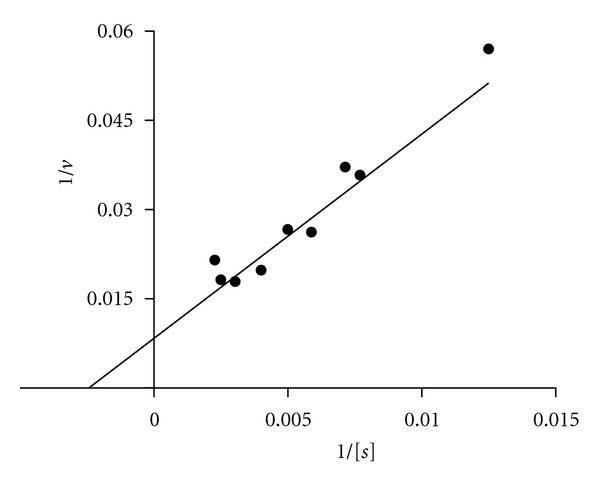
Lineweaver-Burk plot of 1/*v* as a function of 1/[s]. Substrate V was used at concentrations of 0.08 to 0.4 mM. The kinetic parameters were calculated by nonlinear regression analysis using GraphPad software.

The enzyme reaction on 4-nitrophenyl-2-*O*-(methyl-4-*O*-methyl-*α*-D-glucopyranosyluronate)-*β*-D-xylopyranoside (compound V) was quantified using HPLC, and the peak retentions were 14.5 min and 8.4 min for the ester substrate and the acid product, respectively. The kinetic values were determined with *K*_*m*_ 0.25 mM, *V*_max⁡_ 16.3 *μ*M·min^−1^, and *k*_cat_ 9.27 s^−1^ (Figure 7).

## 4. Discussion

The *S. commune* GE gene was constitutively expressed and secreted from *Pichia pastoris* in soluble active form using the pGAPZ vector expression system. The pGAPZ vector consists of the strong promoter of the glyceraldehyde-3-phosphate dehydrogenase gene, fused to an N-terminal peptide encoding the *Saccharomyces cerevisiaeα*-factor secretion signal. The use of methanol inducible vectors with various gene-vector constructions all resulted in negligible expression. It was also found that for the purification of the recombinant enzyme, a quick clarification of the culture by polyethylene sulfone filters followed by membrane ultrafiltration enhanced affinity binding in the Ni-Sepharose column and the recovery of the enzyme.

The structure of *Sc*GE revealed by homology modeling shows a typical hydrolase *α*/*β* fold, as recently determined for the crystal structure of *H. jecorina *enzyme [13]. The molecule is structurally related to the BVU-4111 esterase-like protein found in *Bacteroides vulgatus* (3g8y). The BVU-4111 protein structure is similar to *Bacillus pumilus* AXE (3fvt), *Thermotoga maritima* AXE, and *Bacillus subtilis* cephalosporin C deacetylase (1ods), which are all CE7 serine-type esterases. However, sequence alignment reveals no obvious similarity between the GE enzymes and known AXEs.

The r*Sc*GE does not contain a CBM. This is in contrast to the native GE enzymes isolated from other microorganisms, including *H. jecorina*, *P. chrysosporium*, and *S. thermophile*, which consist of a carbohydrate-binding type 1 (CBM1) domain at the N-terminus linked to the catalytic domain [7–9]. The existence of CBMs has been observed in various carbohydrases, including cellulases and hemicellulases, but not all carbohydrate active enzymes have acquired this type of noncatalytic domain. The most recognized function of CBM is to facilitate the hydrolysis of insoluble polysaccharides. CBM1 structures typically consist of 4 conserved cysteines forming disulfide bonds (http://www.CAZY.org/). The number of aromatic residues and their precise spatial arrangement in the flat face of the type I CBD fold are critical for specific binding with the aromatic rings stacked onto the glucose ring of the cellulose structure [14]. It is not certain at present how the lack of a CBM in the r*Sc*GE enzyme would influence its biochemical action on insoluble substrates. 

The glycosylation of r*Sc*CE was confirmed by N-glycosidase treatment followed by SDS-PAGE. Two potential *N*-glycosylation sites are found at positions 103–106 (NNSI) and 168–171 (NASA) of the sequence. Glycosylation may potentially improve enzyme stability as reported in the literature for some enzymes [15, 16]. The native *Sc*GE that was estimated to be 44 kD did not seem to be glycosylated and had a pI of 3.5 [6]. The native enzymes isolated from *H. jecorina* and *P. chrysosporium* were not glycosylated, although putative glycosylation sites were identified in the sequences [7, 8]. The glycosylation of the recombinant enzyme in the present study may well be the result of the posttranslational processing by the *Pichia* expression system. The acidic pI observed for r*Sc*GE and also for the native enzyme is in contrast to the other GE enzymes, which have pIs in the basic range. The significance of this disparity in the pI requires further investigation.

The r*Sc*GE showed similar action and kinetics on the substrates with functional properties representative of the native enzyme purified from *S. commune*. The r*Sc*GE hydrolyzed all the substrates containing D-glucuronic acid moiety. It did not hydrolyze methyl D-galactopyranuronate (substrate IV), suggesting the importance of the glucoconfiguration at C4 in substrate recognition. The result indicates that the enzyme recognized alkyl and arylalkyl esters of methyl-D-glucuronic acid only. It also provides indirect support that the enzyme specificity was on the ester bonds in substrates with mimics between glucuronoxylan and lignin alcohols. Unlike synthetic mimics, however, the ester bond in the natural substrate formed between the lignin alcohol and the C6 carboxylic group of the methylglucuronic acid entails a very bulky molecule. The synthetic substrates are close but not exact representation of the natural substrate. The physiological role of the enzyme on natural substrates has yet remained to be demonstrated. A recent work on the genome of *Teredinibacter turnerae* T7901 has identified a gene cluster of GH11-CBM5-GE15 encoding a multicatalytic enzyme with the GE15 glucuronoyl esterase combined with GH11xylanase, suggesting its important function in plant cell wall degradation [17]. It is envisioned that a large dose of the enzyme may be required to hydrolyze ester linkages between hemicellulosic uronic acids and lignin alcohols existing in large molecules in plant cell wall. The enzyme may also require collective action of other plant cell wall hydrolytic enzymes. The heterologous expression and production of active r*Sc*CE will facilitate further investigation in this direction.

## References

[1] Wyman CE, Dale BE, Elander RT (2009). Comparative sugar recovery and fermentation data following pretreatment of poplar wood by leading technologies. *Biotechnology Progress*.

[2] Lawoko M, Henriksson G, Gellerstedt G (2005). Structural differences between the lignin-carbohydrate complexes present in wood and in chemical pulps. *Biomacromolecules*.

[3] Painter TJ (1983). Residues of d-*lyxo*-5-hexosulopyranuronic acid in *Sphagnum holocellulose*, and their role in cross-linking. *Carbohydrate Research*.

[4] Imamura T, Watanabe T, Kuwahara M, Koshijima T (1994). Ester linkages between lignin and glucuronic acid in lignin-carbohydrate complexes from *Fagus crenata*. *Phytochemistry*.

[5] Watanabe T, Ohnishi J, Yamasaki Y, Kaizu S, Koshjima T (1989). Binding-site analysis of the ether linkages between lignin and hemicelluloses in lignin-carbohydrate complexes by DDQ-oxidation. *Agricultural and Biological Chemistry*.

[6] Špániková S, Biely P (2006). Glucuronoyl esterase—novel carbohydrate esterase produced by *Schizophyllum commune*. *FEBS Letters*.

[7] Li XL, Špániková S, de Vries RP, Biely P (2007). Identification of genes encoding microbial glucuronoyl esterases. *FEBS Letters*.

[8] Ďuranová M, Špániková S, Wösten HAB, Biely P, De Vries RP (2009). Two glucuronoyl esterases of *Phanerochaete chrysosporium*. *Archives of Microbiology*.

[9] Vafiadi C, Topakas E, Biely P, Christakopoulos P (2009). Purification, characterization and mass spectrometric sequencing of a thermophilic glucuronoyl esterase from *Sporotrichum thermophile*. *FEMS Microbiology Letters*.

[10] Ďuranová M, Hirsch J, Kolenová K, Biely P (2009). Fungal glucuronoyl esterases and substrate uronic acid recognition. *Bioscience, Biotechnology and Biochemistry*.

[11] Hirsch J, Langer V, Koóš M (2005). Synthesis and molecular structure of methyl 4-*O*-methyl-*α*-D- glucopyranuronate. *Molecules*.

[12] Arnold K, Bordoli L, Kopp J, Schwede T (2006). The SWISS-MODEL workspace: a web-based environment for protein structure homology modelling. *Bioinformatics*.

[13] Pokkuluri PR, Duke NEC, Wood SJ (2011). Structure of the catalytic domain of glucuronoyl esterase Cip2 from *Hypocrea jecorina*. *Proteins: Structure, Function and Bioformatics*.

[14] Mattinen ML, Kontteli M, Kerovuo J (1997). Three-dimensional structures of three engineered cellulose-binding domains of cellobiohydrolase I from *Trichoderma reesei*. *Protein Science*.

[15] Olsen O, Thomsen KK (1991). Improvement of bacterial *β*-glucanase thermostability by glycosylation. *Journal of General Microbiology*.

[16] Clark SE, Muslin EH, Henson CA (2004). Effect of adding and removing N-glycosylation recognition sites on the thermostability of barley *α*-glucosidase. *Protein Engineering, Design and Selection*.

[17] Yang JC, Madupu R, Durkin AS (2009). The complete genome of *Teredinibacter turnerae* T7901: an intracellular endosymbiont of marine wood-boring bivalves (shipworms). *PLoS ONE*.

